# Concerning the First Rib

**Published:** 1914-05-02

**Authors:** 


### Concerning the First Rib.

One of the hypotheses that have been advanced to account for the great preponderance of apical tuberculosis of the lungs over phthisis in other parts ascribes an important influence to the first rib in determining the seat of invasion by the tubercle bacillus. In the *British Journal of Surgery* Mr. Morriston Davies publishes the result of his investigations into this indictment of the first rib, which amounts to a verdict of not guilty. He finds that neither abnormal shortness nor ossification of the first costal cartilage predisposes to apical pulmonary tuberculosis. Abnormal shortness of the first costal cartilage does not encourage ossification in that cartilage; such ossification is dependent on age and sex, and probably on occupation. With increasing age there is increasing limitation of movement of the sternal angle; but limitation of this angle does not predispose to apical pulmonary tuberculosis. The presence of a groove in the posterior external aspect of the lung below the apex is not the result of abnormal shortness of the costal cartilage, but probably of emphysema. The formation of a false joint in the rigid cartilage does not tend to lead to the cure of apical tuberculosis; and therefore the balance of evidence is against the probability of the operation for division of the first costal cartilage in cases of apical tuberculosis producing any material improvement.

